# Aging in place among adults with traumatic personal experiences and the community’s role: A scoping review

**DOI:** 10.1093/geroni/igaa057.3225

**Published:** 2020-12-16

**Authors:** Ramona Danielson, Susan Ray-Degges

**Affiliations:** 1 North Dakota State University, Fargo, North Dakota, United States; 2 Interior Design Educators Council (IDEC), Fargo, North Dakota, United States

## Abstract

We propose that trauma history is important context for understanding the adaptive capacity of aging adults to achieve a suitable person-environment fit. We conducted a scoping review using bibliographic databases to identify studies focused on aging in place, vulnerability and traumatic personal experiences, and aging adults’ maladaptive behaviors. Our review showed little research directly exploring the connection between trauma-related needs and aging in place and limited research about the community’s role in supporting those needs. Literature about the impacts of trauma revealed that trauma could stem from adverse childhood experiences (e.g., abuse, neglect, household dysfunction) and adult adverse experiences (e.g., domestic violence, substance use disorder, military trauma). Adults who have experienced trauma may have increased physiological (e.g., sensory sensitivities) and psychological (e.g., post-traumatic stress disorder) conditions. Sensory sensitivities in the home environment—including sounds (e.g., air conditioners), air quality (e.g., pollutants), and lighting (e.g., flickering lights)—can substantially decrease quality-of-life. Strong emotional reactions can make interpersonal relationships, such as those with a landlord or neighbors, difficult and increase the likelihood of eviction. Aging adults with trauma histories also experience greater poverty rates, increasing the likelihood of substandard housing (e.g., mold, rats). Because the housing environment is a significant aspect of overall well-being, challenges to aging in place for adults with trauma histories warrants further research, as does the role of community supports in mitigating housing-related stressors. Examples may include trauma-informed approaches in universal design, the availability of safe affordable housing, community education, and funding for intermediaries who can support aging adults.

